# Detection of cytokines in nasal lavage samples of patients with cystic fibrosis: comparison of two different cytokine detection assays

**DOI:** 10.1186/s12890-024-03103-9

**Published:** 2024-06-19

**Authors:** Teresa Fuchs, Manuela Zlamy, Thomas Zöggeler, Dorothea Appelt, Katharina Niedermayr, Anja Siedl, Verena Gasser, Johannes Eder, Helmut Ellemunter

**Affiliations:** 1 Department of Pediatrics III, Cystic Fibrosis Centre Innsbruck, Medical University of Innsbruck, Innsbruck, Austria; 2 Tiroler Gesundheitsfond, Amt der Tiroler Landesregierung, Innsbruck, Austria; 3 Department of Pediatrics I, Medical University of Innsbruck, Innsbruck, Austria; 4 Department of Pediatrics I, Cystic Fibrosis Centre Innsbruck, Tirol Kliniken, Innsbruck, Austria; 5 Medical University of Innsbruck, Austria, Medical Research Affiliate, Innsbruck, Austria

**Keywords:** Cystic Fibrosis, Inflammation, Cytokines, Nasal lavage, Biomarkers

## Abstract

**Background:**

Cystic fibrosis (CF) is a genetic multisystem disorder. Inflammatory processes, which presumably begin early in infancy, play a crucial role in the progression of the disease. The detection of inflammatory biomarkers, especially in the airways, has therefore gained increasing attention. Due to improved treatment options, patients with CF produce less sputum. Nasal lavage samples therefore represent a promising alternative to induced sputum or bronchoalveolar lavage specimens. However, methodology of cytokine measurements is not standardised and comparisons of results are therefore often difficult. The aim of this study was to identify suitable detection methods of cytokines in nasal lavage samples by comparison of two different assays.

**Methods:**

Nasal lavage samples were obtained from the same patient at the same time by trained respiratory physiotherapists using a disposable syringe and 10 ml of 0.9% sodium chloride per nostril during outpatient visits. The cytokines IL-17 A, IL-2, IL-6 and IL-10 were measured using two different assays (BD™ and Milliplex®), which have already been applied in sputum and nasal lavage samples, despite different lower detection limits.

**Results:**

22 participants were included in the study. In 95.5% of measurements, values were below the limit of detection with respect to the BD™ assay. Only IL-6 could be detected in approximately half of the patients. Individual cytokine levels were considerably higher when measured with Milliplex®, which is also reflected in a statistically significant manner (p = < 0.01).

**Conclusion:**

The right choice of analysis method is crucial for measuring inflammatory markers in nasal lavage samples. Compared to the literature, Milliplex® showed higher detection rates and similar concentrations to other studies.

**Trial registration:**

Ethics approval was obtained from the ethics committee at Medical University of Innsbruck (EK Nr: 1055/2022).

## Introduction

Cystic fibrosis (CF) is a genetic multisystem disorder caused by mutations in the *CF transmembrane conductance regulator (CFTR) gene* [1]. Neutrophil-dominated inflammation of CF airways starts early in life, even without concurrent evidence of bronchopulmonary bacterial burden, leading to a vicious cycle of airway obstruction, infection and progressive inflammation that continues to drive the disease [2]. Quantitative measurement of airway inflammation is therefore important to monitor disease progression. Research in finding adequate biomarkers has gained much interest in recent years [3–5].

Initially, studies focused on the investigation of inflammatory markers in bronchoalveolar lavage (BAL) samples, which were limited by bronchoscopic procedural risks and lack of reproducibility [2, 6]. Alternatively, induced sputum samples, as a noninvasive sample collection method, have gained attention [4, 5]. Another noninvasive option for the analysis of cytokines is with nasal lavage samples (NL) [7, 8]. The collection of sputum proves to be more difficult, especially after introduction of CFTR modulators into routine therapy; therefore, the collection of NL soon became a very promising and simple method to obtain samples for further analysis. Collection can be simply performed during outpatient visits or in a home-based setting, and they can be frozen without complex pre-treatment [7].

Determination of inflammatory markers in the airways of CF patients serves as a clinical outcome parameter, is used in clinical trials and is a target for drug development. Recent studies have focused on the following biomarkers: neutrophil elastase (NE), TNF-α, IL-1β, IL-6, IL-8 and IL-17 [9, 10]. Specifically, NE, IL-8, TNF-α and IL-1β demonstrated validity and are currently considered for use as endpoints in clinical trials [10].

Despite the great efforts put into research on inflammatory makers in recent years, the translation of biomarkers into wider use remains difficult. This is partly due to different sample media used as well as different measurement methods. Therefore, it is difficult to compare individual study results.

Our study aimed to compare the results of two widely used cytokine detection assays from BD™ and Milliplex® in NL samples and to identify the most sensitive measurement method.

## Materials and methods

This study is a subgroup analysis of the INFLAM-CF study (Airway inflammation in patients with cystic fibrosis: immunological markers in sputum and/or nasal lavage in a longitudinal course), conducted at the CF centre Innsbruck, Austria (CFCI). Patients were included between April and June 2022. Study visits and sample collection were conducted during regular outpatient visits. Inclusion criteria were as follows: confirmed diagnosis of CF (identification of at least one disease-causing variant and/or 2 positive sweat tests) and stable disease without treatment changes 4 weeks before enrollment. Exclusion criteria included therapy with systemic or inhaled steroids 4 weeks prior to inclusion, general use of immunosuppressive therapy and chronic bronchopulmonary non-tuberculous mycobacteria colonization, pulmonary exacerbations (defined according to modified Fuchs criteria [11]), upper respiratory tract infections, and the receipt of any vaccinations 4 weeks prior to inclusion. None of the included patients had a known history of sinusitis, presence of polyps, or concomitant allergic rhinitis.

Written informed consent was obtained from each patient and/or their legal guardian. The study was approved by the local ethics committee (EK Nr: 1055/2022). None of the companies mentioned acted as sponsors of this study; no funding was received from BD™ or Milliplex®.

### Study population

Our study was performed as a cross-sectional study. 22 participants from the CFCI were included. Of these, 50% were female (11 male), and 50% were pediatric patients. At screening visit, patients were aged 3–36 years (mean age 18 years). 50% of all patients were considered chronically colonized (3 pediatric, 8 adult), including 4 with chronic *Pseudomonas aeruginosa* colonization (adult only) and 4 with colonization by *Staphylococcus aureus* (2 pediatric, 2 adult) according to Leeds criteria [12]. Other chronic pathogens included *Aspergillus fumigatus complex*, *Stenotrophomonas maltophilia* and *Burkholderia cepacia complex*. 6 patients used sustained inhaled antibiotic therapy for several weeks prior to our study (1 pediatric, 5 adult). 7 patients were treated with CFTR modulators at screening, of whom 5 received highly effective modulator therapy (HEMT). Patient characteristics are shown in Table 1.

**Table 1:** Patient demographics

Variable	Result
n (%)	22
Pediatric	11 (50%)
Adult	11 (50%)
Gender n (%)	
male	11 (50%)
female	11 (50%)
Age at screening (y) mean (range)	18 (3–36)
Pediatric	9.8 (3–16)
Adult	26.3 (18–36)
CFTR genotype n (%)	
dF508 homozygous	7 (31.8%)
dF508 heterozygous	13 (59.1%)
Other	2 (9.1%)
HEMT therapy at screening n (%)	5 (22.7%)
Pediatric	0 (0%)
Adult	5 (45.5%)
ppFEV_1_ (%) mean (range)	89.5 (54.4-121.5)
Pediatric (*n* = 10)	89 (75.8-105.6)
Adult	89.9 (54.4-121.5)
Lung clearance index mean (range)	7.6 (5.5–11.8)
Pediatric	6.9 (5.6–8.9)
Adult	8.3 (5.5–11.8)

### Nasal lavage collection

NL collection was performed at screening visits by trained respiratory physiotherapists. First step involved gentle cleansing of the nose. Then a disposable syringe was used to insert 10 ml of 0.9% sodium chloride per nostril by extending the neck approximately 30° from the horizontal or in axis-correct oblique posture of the upper body and head in infants. The daily performance of nasal lavage is recommended in our centre from the time of diagnosis (usually in the second month of life) and its correct implementation is regularly checked by our respiratory physiotherapists as part of outpatient clinics. Our patients, including infants, are therefore used to this procedure. All patients, regardless of age, received the same amount of 0.9% sodium chloride lavage. The average sample return volume was 15 ml. NL fluid was immediately aliquoted into disposed reaction tubes of 2.5 ml sample volume each. Reaction tubes contained 15 µl of protease inhibitor (Protease Inhibitor Mix G, SERVA®) and were frozen at -80 °C until further analysis as described in literature [7]. Since we did not expect any significant cell debris in the nasal lavage procedure, the centrifugation step was skipped.

### Cytokine analysis

Concentrations of IL-17 A, IL-2, IL-6 and IL-10 were measured in undiluted NL fluid obtained at the same time from the same patient. Two different detection assays were each applied to all 22 patient samples. The aliquots were completely thawed for at least 30 min before further analyses were performed.

One detection assay used was the BD™ Cytometric Bead Array (BDA) System, which uses bead array technology and flow cytometric analysis (BD Life Science – Bioscience®, San Jose, USA). The tests were performed according to the manufacturer´s instructions. Each bead had a distinct fluorescence and was coated with a specific antibody. If certain analytes were present in the nasal lavage, a fluorescence signal was generated which was proportional to the amount of bound analytes. The result was a sandwich complex (capture bead + analyte + detection reagent) which was subsequently measured by flow cytometry. As specified, the minimum detection limits were 18.9 pg/ml (IL-17 A), 2.6 pg/ml (IL-2), 2.4 pg/ml (IL-6) and 4.5 pg/ml (IL-10) respectively. After flow cytometric analysis, FCAP Array ^TM^ software, V3.0. (Soft Flow Ltd. Hungary) was used for quantification of cytokine concentrations.

As a second assay, we used Milliplex MAP-Kits® (Human Cytokine/Chemokine/Growth Factor Panel A, Merck Millipore, Darmstadt, Germany), which uses magnetic bead load based on Luminex® technology and a MAGPIX® system (Luminex Corporation, Austin, USA) according to the manufacturer´s instructions. The technique used two fluorescent dyes, which create distinctly coloured beads of magnetic microspheres. Each of the beads was coated with a specific capture antibody. If analytes in the sample were captured by the bead, a detection antibody was introduced. This reaction mixture was then incubated with Streptavidin-PE conjugate. As specified by the manufacturer, the minimum detection limits were 0.71 pg/ml (IL-17 A), 0.28 pg/ml (IL-2), 0.14 pg/ml (IL-6) and 0.91 pg/ml (IL-10). Measurements were performed in duplicate.

### Statistical analysis

Statistical analysis was performed using SPSS® Statistics Software 27 for Windows (IBM Corporation, Armonk, USA). Kolmogorov‒Smirnov test was used to determine a normal distribution, Wilcoxon test was used as a nonparametric test. Nonmeasurable values in the BD™ kit were assigned a value of 0 pg/ml. P values equal to or smaller than 0.05 were defined as statistically significant.

## Results

Determination of cytokine levels was performed in July and August 2022, respectively. At the time of sample collection, all patients presented in stable health without current or recent respiratory infection. Focusing on the assay-specific detection rate, the BD™ assay showed detection of IL-17 A, IL-2 and IL-10 in only 4.5% of tests. 95.5% of samples had undetectable levels of 0 pg/ml. IL-6 was slightly elevated in 14 patients (63.6%) with mean values of 1.3 pg/ml (range 0-8.7 pg/ml).

**Fig. 1:**
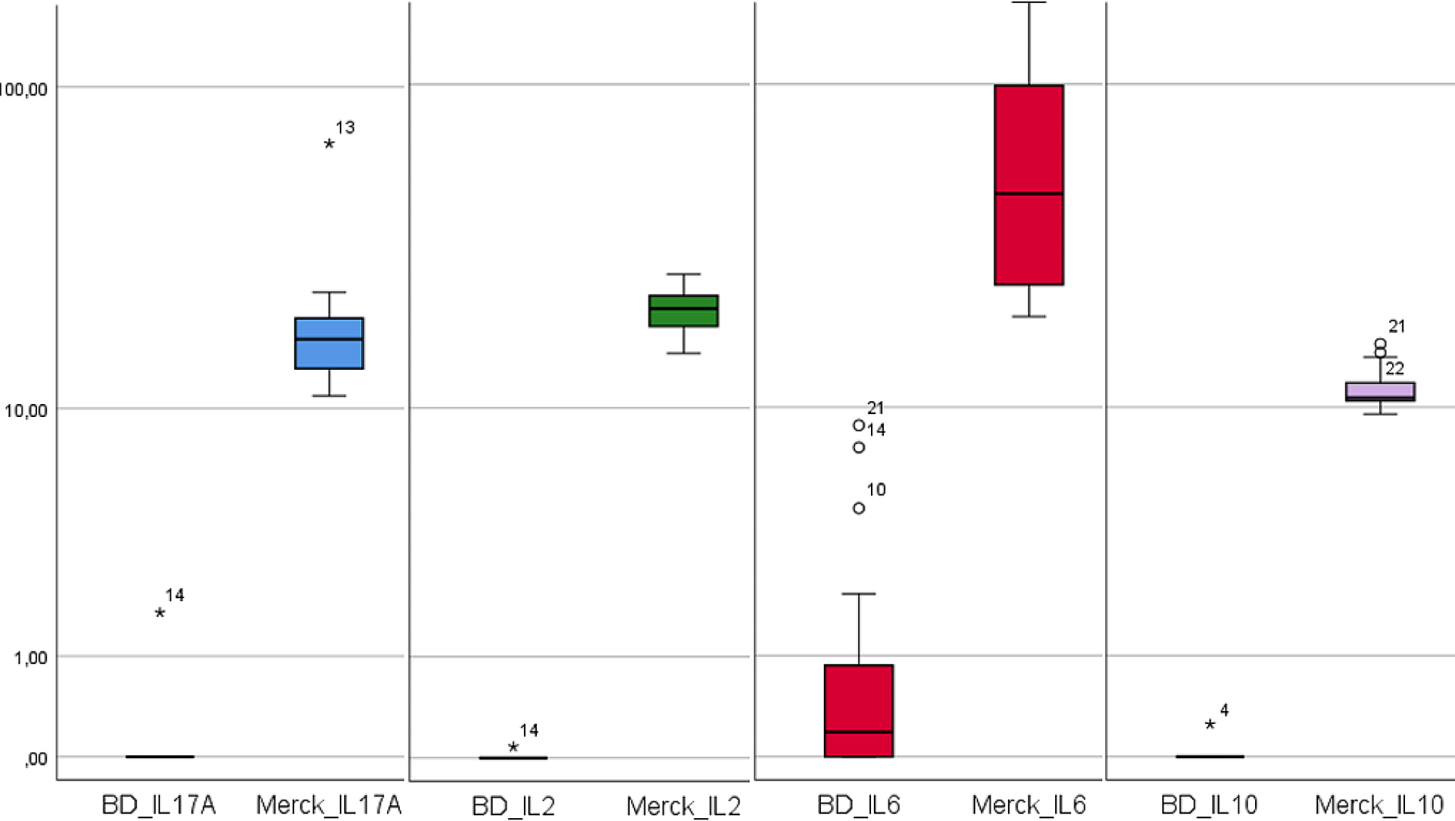
Boxplots of IL-17 A, IL-2, IL-6 and IL-10 measured with BD™ and Milliplex ®. Wilcoxon test was used as non-parametric test. P values equal to or smaller than 0.05 were defined as statistically significant. BD = BD™; Milliplex = Milliplex®.

Cytokines were detectable in 100% of tested NL samples using the Milliplex® assay. Individual cytokine levels were considerably higher when measured with Milliplex®, which is also reflected in a statistically significant manner (p = < 0.01) for each value. Statistical results are shown in Table 2; Fig. 1.

**Table 2:** Statistical analysis

Statistics			
Variable	BD™ mean pg/ml *n* = 22	Milliplex® mean pg/ml *n* = 22	*P* value
IL-17 A	0.077 (0-1.7)	18.682 (11.0-67.25)	**< 0.01** ^**a**^
IL-2	0.004 (0-0.08)	20.545 (15.0-26.5)	**< 0.01** ^**a**^
IL-6	1.255 (0-8.72)	73.068 (19.5-290.75)	**< 0.01** ^**a**^
IL-10	0.011 (0-0.25)	11.432 (9.5–16.0)	**< 0.01** ^**a**^

^a^ by Wilcoxon-Test

## Discussion

Biomarkers are used as outcome parameters in clinical trials to reflect airway inflammation. However, when we look at the wide range of biomarkers that have been explored to date, it is still difficult to filter those that might serve best as surrogate markers to predict clinical outcomes. Similarly, there is wide variability in sample selection and cytokine detection methodology used. Looking at data regarding the effect of CFTR modulators on inflammation, a divergent picture emerges thus far [13–15]. Therefore, the identification of suitable biomarkers as targets for anti-inflammatory therapy remains important despite HEMT. The establishment of a unification of methodological measures is important to compare study results and facilitate the exploration of appropriate biomarkers. The aim of our study was to simplify the selection of appropriate methods. For this purpose, the additional evaluation of clinical parameters was intentionally omitted to focus on the precise elaboration of technical exercise.

In the era of HEMT, patients with CF experience higher quality of life as well as lower disease-related morbidity [16]. The decreasing amount of sputum due to this therapy and the generally low amount produced in children have complicated the analysis of inflammatory biomarkers in sputum samples. Therefore, an alternative sample material collection method such as NL could be very helpful.

The detection assay by BD™ was also used in a study by Eckrich et al. in patients with mild CF and healthy controls [17]. The cytokines/chemokines IL-1β, IL-6, IL-8 and TNF-α were analyzed in induced sputum samples. In particular, IL-1β and IL-8 showed higher values at 800–900 pg/ml and 10.000-100.000 pg/ml, respectively (data by Eckrich et al. are only shown in figures, numerical data are not available) [17]. An older study conducted by Kumar et al. analyzed IL-4, IL-6, IL-8, IL-1β, TNF-α and IFN-γ in BAL samples from patients with suspected ventilator-associated pneumonia without CF as an underlying disease. Additionally, this study showed high values of IL-6 (mean 5000 pg/ml) and IL-8 (mean 22.800 pg/ml) measured by BD™ [18]. In both of these studies, much higher cytokine levels were measured, although Eckrich et al. included patients with CF presenting with mild disease progression compared to our data. Criteria for mild disease included forced vital capacity (FVC) > 75% and forced expiratory pressure in 1 s (FEV_1_) > 70%. Comparison of ppFEV_1_ values showed a mean of 93.6% in Eckrich et al. compared to 89.5% in our cohort [17]. Thus, it appears that our patients have worse lung function in the sense of advanced lung disease and, at the same time, a lower cytokine load. In 95.5% of our patients, IL-17 A, IL-2 and IL-10 could not be detected in NL samples using the assay by BD™.

The assay by Milliplex® has already found application in NL samples. Erdmann et al. studied NL samples from patients with CF, showing mean values of 15.7 pg/ml (IL-6), 203.3 pg/ml.

(IL-8) and 4.3 pg/ml (IL-1β) [8]. Similar concentrations were also measured by Mainz et al. using Milliplex® in NL samples [19]. These results are comparable to our values. Another study by Mulvanny et al. used a Milliplex MAP Human High Sensitivity T-Cell Panel and analyzed cytokines in sputum samples in patients with stable chronic obstructive pulmonary disease (COPD) [20]. This study showed values below 400 pg/ml for IL-6, which are on average higher than IL-6 measured in NL [8, 18, 20].

The main reason for the measurement discrepancies in the BD™ kit between our cohort and the results of Eckrich et al. and Kumar et al. are certainly the different sample materials. However, our study shows that this kit may be applicable for induced sputum and BAL but not for nasal lavage samples.

Looking at the direct comparison of the two assay kits used in our study, clear differences in concentration can be seen, illustrated in IL-2 (0.004 vs. 20.545 pg/ml, p = < 0.01) and IL-6 (1.255 vs. 73.068 pg/ml, p = < 0.01). A strength of our study is that the same material from the same patient at the same time of collection was examined with two different methods. We are therefore able to present a direct comparison.

Major limitations in our study include the small sample size and single-centre study format. It must also be mentioned that BD™ exhibited a considerably higher limit of detection than Milliplex ®. This discrepancy likely contributes to the observed differences in results.

In summary, our results confirm the basic problem of finding suitable methods for the measurement of cytokines in patients with CF. There are no data on the direct comparison of cytokine levels measured in the same samples yet. Our study, however, shows a direct comparison of two measurement methods in the same patient. Due to the different lower detection limits described in the individual assay manuals, different measurement results were to be expected. However, our study emphasizes the importance of careful selection of assay kits. Concerning NL samples, which probably have a very promising future with easy sample collection and processing, the Milliplex® assay is the better choice when measuring inflammatory markers. For further comparison of cytokine detection assays, a larger study population must be analyzed.

## Data Availability

No datasets were generated or analysed during the current study.

## Abbreviations

CF: Cystic Fibrosis
CFTR: CF transmembrane conductance regulator
BAL: Bronchoalveolar lavage
NL: Nasal lavage
NE: Neutrophile elastase
CFCI: CF centre Innsbruck
HEMT: Highly effective modulator therapy
